# Talking Parents, Healthy Teens: A Worksite-based Program for Parents to Promote Adolescent Sexual Health

**Published:** 2006-09-15

**Authors:** Mark A Schuster, Karen L Eastman, Rosalie Corona

**Affiliations:** UCLA/RAND Center for Adolescent Health Promotion. Dr Schuster is also affiliated with the Department of Pediatrics, David Geffen School of Medicine at UCLA, Los Angeles, Calif, the Department of Health Services, UCLA School of Public Health, Los Angeles, Calif, and RAND, Santa Monica, Calif.; David Geffen School of Medicine at UCLA, Los Angeles, Calif; Department of Pediatrics, David Geffen School of Medicine at UCLA and Virginia Commonwealth University, Richmond, Va

## Abstract

Parents play an important role in the sexual health of their adolescent children. Based on previous research, formative research, and theories of behavioral change, we developed Talking Parents, Healthy Teens, an intervention designed to help parents improve communication with their adolescent children, promote healthy adolescent sexual development, and reduce adolescent sexual risk behaviors. We conduct the parenting program at worksites to facilitate recruitment and retention of participants. The program consists of 8 weekly 1-hour sessions during the lunch hour. In this article, we review the literature that identifies parental influences on adolescent sexual behavior, summarize our formative research, present the theoretical framework we used to develop Talking Parents, Healthy Teens, describe the program's components and intervention strategies, and offer recommendations based on our experiences developing the program. By targeting parents at their worksites, this program represents an innovative approach to promoting adolescent sexual health. This article is intended to be helpful to health educators and clinicians designing programs for parents, employers implementing health-related programs, and researchers who may consider designing and evaluating such worksite-based programs.

## Introduction

As documented by the Centers for Disease Control and Prevention's (CDC's) Youth Risk Behavior Survey (YRBS), many adolescents engage in behaviors that increase their risk of sexually transmitted diseases (STDs) and unintended pregnancies (1). Most efforts to promote healthy adolescent sexual development and reduce risk have targeted adolescents through community- or school-based programs (2-5). There has been much less focus on the protective role parents can play in raising sexually healthy adolescents.

We developed Talking Parents, Healthy Teens, a program to help parents learn parenting and communication skills that would facilitate communication with their adolescent children, promote healthy adolescent sexual behaviors, and reduce sexual risk behaviors. The program is provided at worksites as a means of reaching a large number of parents easily.

In this article, we briefly review the role that parents can play in adolescent sexual health, present the theoretical framework used to develop Talking Parents, Healthy Teens, and describe the program's components and intervention strategies.

## Background

Certain parenting behaviors and types of parent–adolescent relationships are related to adolescent risk behaviors. For example, adolescents whose parents monitor them are more likely than others to initiate intercourse at later ages (6-8) and to have fewer partners and use condoms if they are sexually active (9-12). The more involved parents are with their adolescents (e.g., knowledgeable about their school and extracurricular activities), the less likely their adolescents will be to initiate sex at earlier ages and to engage in drug use and other problem behaviors (13-15). In addition, adolescents are less likely to initiate intercourse at a young age or engage in frequent intercourse, and more likely to use contraception, if they are positively connected to their parents (e.g., feel satisfied in their relationships) (16-18).

Although older studies on the relationship between parent–adolescent communication and adolescent sexual behavior have shown mixed results (19-21), some researchers have found that when parents talk to their adolescents about sexuality, adolescents are more likely to delay intercourse and if they have intercourse, to use contraception and have fewer partners (22-24). Yet, many parents do not feel comfortable talking with their adolescents about sexual topics (25); when parents talk about these topics, they tend to lecture (26), possibly inhibiting open communication. Parents who feel more confident in their parent–adolescent communication skills are more likely than less confident parents to engage in conversations about sex (27-29). In addition, parents' use of open-ended questions is positively associated with adolescent engagement in conversations about sexuality (30).

Despite the evidence for the protective role of parents in adolescent sexual health, most HIV and sex education programs targeted at teens have no role or a limited role for parents (4). Although these programs are an important component of health promotion efforts for youth, their effects often extinguish fairly rapidly. By contrast, programs that help parents influence their adolescents' behaviors may have more enduring effects. Parents generally have more contact than most other adults with their adolescents, are familiar with their adolescents' attitudes and idiosyncrasies (or could be), and are invested in their children's lives. Given parents' long-term perspective on the implications of their adolescents' sexual health and development and their ability to retain and use knowledge and skills, parents have the potential to provide the ongoing reinforcement that time-limited youth programs can rarely offer. As a result, there has been a push to develop parent-only programs (31-33), but few have actually been evaluated (26,34,35), and others are undergoing evaluation (36). Our program adds to this growing number of parenting programs but is unique in that it is the only such program that we know of that is delivered at a parent's workplace and is undergoing rigorous evaluation in a randomized controlled trial.

## Strategies

### The worksite setting

Interventions aimed at parents need to reach and engage them. This can be difficult in community settings where many parents must make a special effort to attend (37). Parent training programs on various topics generally have high dropout rates, ranging from about 25% to more than 40% (38,39). A promising alternative is to bring the intervention to parents where they work, an approach that may facilitate recruitment and retention (40). Worksite-based health programs, such as weight reduction (41) and smoking cessation (42), have been successful in changing employees' health-related behaviors. Although some employers have programs to help employees with family issues, few have programs designed to address the needs of parents of adolescents. Talking Parents, Healthy Teens addresses this gap.

Additional advantages of the worksite setting include having the support of the workplace management, which can serve as a form of "approval" that makes the parenting program more inviting to employees. Finally, worksites may provide an infrastructure that makes them an easier setting than others for implementing Talking Parents, Healthy Teens or similar programs. 

### Formative research: curriculum development

In developing the parenting program, we 1) reviewed and adapted curricula of parenting programs (general programs and programs covering parent–adolescent communication) and adolescent programs; 2) consulted with researchers and educators with expertise in adolescent behavior, parenting, health promotion, and adult learning principles; 3) conducted focus groups with parents and adolescents and interviews with worksite representatives (43); and 4) piloted the program at three worksites and then revised it based on our experiences.

### Theoretical model

In 1991, the leading proponents of behavior change theories dominating HIV-related research (e.g., social learning theory, health belief model, theory of reasoned action) came to consensus on the eight variables that most strongly influence behavior change (44). They identified three factors as necessary and sufficient: 1) an individual’s *skills* or ability to engage in behavior; 2) an individual’s *intentions* to engage in behavior; and 3) the absence of *environmental barriers* that prevent behavior or the presence of resources (*facilitators*) to engage in behavior. Five additional factors have both a direct and an indirect effect on behavior by influencing intentions: 4) perceived self-efficacy; 5) perceived social norms; 6) perceived net benefits; 7) perceived consistency with personal standards (i.e., behavior is consistent with self-image); and 8) emotional response (i.e., emotional reaction to behavior is more positive than negative). Knowledge and beliefs also influence these five factors.

We applied these eight factors to Talking Parents, Healthy Teens (Figure) and hypothesized that parents would change their parenting behaviors, which would lead to a change in adolescent behaviors. Talking Parents, Healthy Teens aims to influence parents' *skills* such as communication, monitoring, and involvement; *intentions* to talk about sex, monitor, and stay involved; and perceptions of *environmental barriers* and *facilitators* that influence talking about sexuality (e.g., community norms that discourage or encourage such communication). By increasing parents' skills and facilitating opportunities for communication through take-home activities, the program also aims to affect the parent–adolescent relationship, further influencing adolescent behavior change (e.g., the likelihood that adolescents will delay intercourse or use condoms).

**Figure 1:**
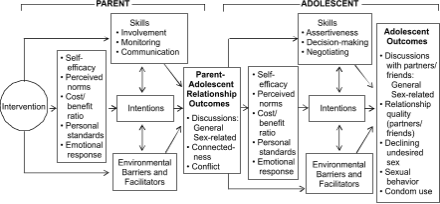
Theoretical model of the relationship between parent–adolescent interactions and adolescent behaviors for the Talking Parents, Healthy Teens program

**Table:** 

Other examples illustrate the types of interactions captured by the theoretical model:

- Parents learn communication skills (e.g., encouraging youth to express their feelings and thoughts) aimed at strengthening their relationships with their children. They also learn communication skills they can teach their children to use in peer and romantic relationships (e.g., assertiveness skills such as how to say no to undesired activities). Parents learn how to improve the parent–adolescent relationship and build on that relationship to teach the child skills that influence behavioral outcomes.
- Parents can monitor adolescents more effectively (e.g., calling home during the afternoon after the child returns from school or arranging for an adult to be home). Parental monitoring can influence adolescent outcomes through the parent–child relationship.
- Improving parent–adolescent communication (e.g., talking about pregnancy prevention) may affect child factors (e.g., ability to negotiate condom use) that influence an adolescent's *intentions* (e.g., to use condoms) and subsequent behaviors (e.g., condom use). The quality of the parent–adolescent relationship may influence how an adolescent responds to a parent's belief about appropriate sexual behavior. For example, if the parent and adolescent have a distant relationship, the child may be more likely to dismiss the parent's view; if the relationship is close, the parent's opinion may influence the adolescent's intentions and behaviors.
- Parents' feelings of self-efficacy and emotional responses may influence their intentions and consequently their communication with their adolescents about sex. For example, parents may feel more competent to talk about sex and therefore more positive about having conversations about sex, which can lead to more frequent and effective communication with their adolescents.

## Program Description: Key Features

### Structure 

Talking Parents, Healthy Teens is a parenting program for parents of sixth to tenth graders. It consists of eight weekly 1-hour sessions presented during the lunch hour to groups of about 15 parents. A trained facilitator and assistant facilitator lead the program using a standardized, scripted, program manual. We provide lunch, which serves as an incentive for participation and reduces late arrivals. The program is interactive and focuses on building parents' abilities, comfort, and confidence; lecturing is minimal. Sessions focus on skill-building and practice. Each session builds on previous ones; the facilitator reviews the prior week's lessons and troubleshoots issues that arose when parents used new skills at home. We mail materials to parents who miss sessions (usually through interoffice mail at the worksite), and the facilitator reviews the session content with absent parents by telephone.

### Diversity of participant values and comfort discussing sex

The program acknowledges that parents have diverse experiences and backgrounds; values, and moral and religious beliefs; and levels of comfort addressing sex-related topics. It is designed so that parents can apply what they learn to achieve their goals. We teach skills, facts, and options and offer advice for how and when to talk to children, but we do not dictate to parents what they should do or how they should feel. For example, to provide balance for parents with diverse views, the same session covers how to say no to sex and how to use a condom. We have had favorable feedback from parents who want their children to refrain from sex until marriage and parents who are comfortable with their high-school-aged adolescents having sex (with contraception).

### Communication skills

Communication skills are a major program feature. For example, parents learn how to start and sustain conversations on sensitive sex-related topics, how to ask questions, and how to listen without lecturing. After parents learn basic communication skills, they learn skills that they can teach their children. The facilitator reviews the elements of each skill and provides examples illustrating its use and benefits. Volunteers read aloud parent–child dialogues that use (or fail to use) the skill, and then all parents practice the skill in role-plays.

### Videotaped role-plays

Between sessions 4 and 7, parents meet individually with the facilitators for a private session to practice the skills and receive feedback. The parent and one of the facilitators, who plays the role of the adolescent based on the parent's description of his or her child, engage in a role-play about a sex-related topic. The role-play is videotaped so that the facilitators can review it with the parent. Parents observe their tone, word choice, and body language in what can be an eye-opening exercise. They then develop a plan to improve their communication.

### Weekly activities 

Each week, parents receive a set of short activities to help them practice new skills at home. Some exercises help parents think about important issues related to their adolescents (e.g., appropriate supervision), and some help parents communicate with adolescents by providing games to play and sex-related topics to discuss (Table).

### Handouts

Parents receive the following handouts during the program: 1) *facts of life*, which cover topics such as puberty, contraception, HIV and other STDs, sexual orientation, and alcohol use; 2) *communication skills*, which summarize communication skills taught during the sessions; 3) *parenting tips*, which provide additional examples of parenting strategies; 4) worksheets, which are used for in-class exercises that help parents learn program material; 5) key ring cards — short outlines of communication skills printed on small laminated cards and attached to a key ring — used so that parents can keep skill summaries handy; and 6) a parenting resource list that includes hotlines, books, and other resources. Parents also receive a participant notebook in which to keep handouts and notes.

### Rewards

Raffles with prizes (e.g., a teen sexual health book) are held during the program. At the end of the program, parents receive a certificate for course completion that provides a marker of their accomplishment and encourages continued work on parent–child relationships.

## Summary of Sessions

### Session 1: Building your relationship with your child

**Overview.** Session 1 provides an overview of the program and reasons for offering it. The session focuses on positive parent–child relationships, covering points that are reinforced in later sessions: the importance of 1) talking to children about sex; 2) establishing a quality parent–child relationship; 3) identifying and reinforcing children's strengths; 4) spending time with children; 5) helping children develop future goals; and 6) supervising children.

**Communication skills.** Parents are encouraged to *praise* or reinforce their children's strengths by "catching their child doing something good" (i.e., noticing a positive behavior and making a favorable comment to the child about it).

### Session 2: Your adolescent's development and new ways of communicating

**Overview.** Session 2 focuses on the importance of being involved in the adolescent's life and reinforces positive parent–adolescent relationships. By discussing adolescent physical, social, emotional, and cognitive development, parents learn that some adolescent behaviors that are baffling and frustrating may be a normal part of development. They are reminded of how physical changes may affect the way adolescents feel about themselves and that an adolescent's sexual and romantic feelings are developing. The topic of sexual orientation is introduced.

**Communication skills. **Parents are introduced to two skills. 1) "*I" messages* are statements parents make that include the phrase, "I feel. . . ." For example, "When you play your music loudly, I feel annoyed because I can't get my work done." These messages do not label or blame the adolescent; they focus on the parent's feelings and not on the adolescent's misbehavior. "I" messages can reduce the likelihood that conflict will escalate. 2) Strategies for *inviting children to talk* (e.g., offering several examples of what a person might feel in a given situation to help adolescents identify and discuss their own feelings) can increase the likelihood of general conversation and may be particularly helpful to parents whose children frequently give responses like "uh huh." The program reinforces the value of having general, nonspecific conversations with adolescents in addition to engaging in specific conversations about sex. The facilitator addresses parents' inability to make children talk if they do not want to and the value of spending time together engaged in activities.

### Session 3: Listening skills for talking about sensitive topics

**Overview.** Session 3 focuses on listening to adolescents and addresses parents' concerns about talking about sex. Parents identify and discuss reasons why they might be reluctant to talk with their children about sex (e.g., fear that talking about sex might encourage it, that the child is too young to talk about it, that they might disclose more about their own past than they want to). By addressing these concerns, parents develop the confidence to talk to their children about sex.

**Communication skills.** Parents learn an approach called *active listening*, which involves paying attention, listening without interrupting, restating what they have heard their children say (to confirm they understood correctly and to show they were listening), and identifying the feelings their children are expressing. Active listening shows youth that parents are interested, encourages youth to express themselves, and helps them identify their own thoughts and feelings. In conversations about sex, this communication skill increases the likelihood that parents and adolescents will engage in a balanced discussion instead of an intervention in which the parents lecture and the adolescents say little.

### Session 4: Talking about sex: getting past roadblocks

**Overview.** In Session 4, the program moves from skills that promote general communication and positive parent–child relationships to skills that support communication specifically about sex. Although many parents have a vague feeling that they do not want their child to have sex, they may not have identified their specific beliefs or considered how they feel about dating and sexual behaviors that might occur before or instead of intercourse. Identifying their beliefs helps parents consider what messages they want to convey.

**Communication skills.** Parents are introduced to four strategies to initiate conversations about sex: 1) using teachable moments (i.e., everyday situations, such as watching a movie with a love scene, that provide opportunities to start discussions); 2) thinking of opening lines to start the conversation; 3) identifying roadblocks (e.g., what adolescents say to make it hard to talk about sex) and strategies such as open-ended questions to get past them; and 4) identifying reasons they want to talk about sex with their children and learning how to avoid lecturing. By practicing how to start conversations through role-plays, parents gain experience and confidence so they can talk to their children more easily.

### Session 5: Helping your child make decisions

**Overview.** Session 5 focuses on developing abilities to engage in longer conversations about sex-related topics with adolescents. Parents think about the reasons that adolescents might and might not want to have sex. By considering the adolescent perspective on sexual matters, parents can anticipate potential adolescent responses and work to make their discussions proceed smoothly.

**Communication skills.** Parents are presented with reasons why it is important to help children learn how to make their own healthy decisions about sexual behavior rather than dictating to them what to do. Parents are introduced to decision-making skills that involve the parent asking the adolescent questions to help the adolescent develop decision-making skills. These decision-making skills are called the *S.T.O.P. steps*: **S**tate the decision; **T**alk about feelings and needs; brainstorm and discuss **O**ptions; and **P**ick the best option and later evaluate it.

### Session 6: Assertiveness skills, abstinence, and contraception

**Overview.** The first part of Session 6 covers assertiveness skills for adolescents who want to remain abstinent from sexual activity in general or refrain from some or all sexual activities in a particular situation. The second part of the session addresses various methods of preventing STDs or unintended pregnancies among adolescents who engage in sexual activity. Parents discuss advantages and disadvantages of condoms and how they would talk to their children about them. The facilitator demonstrates how to use a condom by putting it on two fingers, and parents have the opportunity to practice how they would teach their adolescents the steps for correct condom use.

**Communication skills.** Parents learn assertiveness skills so that they can teach them to their children: how to say no to someone who is applying pressure in an unwanted sexual situation; how to suggest an alternative activity as a means of getting out of a pressured situation without implying a desire to end the relationship (e.g., proposing to go to the movies instead); and delay tactics or methods of cooling down a pressure situation (e.g., going to the restroom). Not only do parents engage in role-plays in which they practice responding to someone who is pressuring them, but they also are encouraged to use these role-plays at home with their adolescents.

### Session 7: More assertiveness skills, coping with conflict, and supervising your kids

**Overview.** Session 7 addresses strategies for negotiating conflict. Parents learn additional assertiveness skills that adolescents can use if they decide to have sex and want to use contraception. Parents review the program skills that can be used to cope with conflict. For example, they are shown how the S.T.O.P. steps from session 5 can be used to resolve problems and reduce conflict with others. Parents also discuss their supervision practices and how to supervise their children appropriately in various situations. Finally, parents discuss what it means to "respect others" and how they can help their children understand concepts such as "no means no."

**Communication skills. **Additional assertiveness strategies that parents learn to teach adolescents include stating that they want to use a condom, giving a reason why they want to use a condom, coming up with a response that they can use if pressured to have sex without a condom, saying no to sex without a condom, and using alternative actions and delay tactics.

### Session 8: Putting it all together and staying motivated

Session 8 reviews the communication and parenting skills learned in the prior seven sessions, motivates parents to continue using these skills, and acknowledges parents' efforts and participation. Parents have the opportunity to practice all of the skills they have learned during the program in a variety of role-plays. They are encouraged to stay in touch with and support each other, to remember to "catch themselves doing something good," and to identify the next conversation they intend to have with their child about sex or sexuality. Finally, rewards for perfect attendance and certificates of participation are distributed. Parents also receive the parenting resource list.

## Evaluation

We are currently conducting a randomized controlled trial of Talking Parents, Healthy Teens, with randomization at the individual parent level. Thirteen worksites in southern California are participating in the evaluation. Worksites include for-profit businesses, nonprofit organizations, and public agencies. The program has been provided to 20 groups of parents, and we are collecting follow-up data. Median attendance was seven out of eight sessions. Feedback has been quite favorable. For example, on a postintervention survey, 96% of participants reported that they would definitely (72%) or probably (24%) recommend the program to a friend or coworker.

## Discussion and Conclusions

Talking Parents, Healthy Teens is a promising approach for improving parenting and communication skills as a means of promoting healthy adolescent sexual development and reducing sexual risk behaviors. Based on theories of behavioral change, Talking Parents, Healthy Teens teaches parenting and communication skills that research suggests are effective. It also includes features characteristic of successful sexual health and HIV prevention programs. Although there seem to be few parenting programs that focus on adolescent sexual health, even fewer have been rigorously evaluated. We are currently evaluating Talking Parents, Healthy Teens' effects on parents and their adolescents.

Our experiences developing this program suggest that 1) parents provide a unique avenue for reaching adolescents; 2) activities and strategies based on adult learning principles can be used to teach parenting and communication skills needed to address many of the challenges parents face in talking to their children about sex; 3) these teaching strategies can engage groups of adults who have various learning styles and parenting and communication abilities; and 4) programs can be designed that are acceptable to parents with diverse values and backgrounds. We recommend that health educators, researchers, and other professionals further explore ways to work with parents to improve the parent–child relationship and to influence adolescents' behavior.

Finally, our preliminary experiences conducting Talking Parents, Healthy Teens at worksites suggest that 1) the worksite setting makes attendance more convenient for many parents of adolescents; and 2) innovative and successful collaborations can occur between clinicians or researchers who are addressing adolescent sexual health and worksite personnel dedicated to improving their employees' family health. We recommend further development of worksite-based programs to address such family issues as adolescent health promotion.

## Figures and Tables

**Table:** Summary of Weekly Activities to Be Completed Outside of Program Sessions: Talking Parents, Healthy Teens Worksite Intervention

**Session**	**Activities**
1	Parents practice catching their adolescent doing something good. Parents play a question-and-answer game with their child to get to know their child's likes and dislikes. Parents identify time periods or situations in their child's daily life when they might want to supervise their child more or grant their child more freedom or independence.
2	Parents spend time with their adolescent child by engaging in a fun activity chosen by the child. Parents and their adolescent play "The Changes Game," a question-and-answer card game designed to provide an opportunity to talk about the physical changes that occur during adolescence. Parents read three descriptions of adolescent behavior and write "I" messages to express how they might feel in each situation.
3	Parents read questions and discuss with their child the qualities their child values in close relationships. Parents read questions and discuss with their child the qualities that are important in healthy romantic relationships. Parents identify messages about sex or sexuality that they would like to communicate to their child.
4	Adolescents interview their parents about what life/dating was like when they were teenagers. Parents brainstorm ways that they might be able to supervise their children more or allow their children greater freedom and independence during the time periods they identified previously. Parents practice their active listening skills with a coworker or friend.
5	Parents use several scenarios and questions to help teach their adolescent decision-making steps. Parents and their adolescents come up with five reasons why adolescents have sex or want to have sex and five reasons why adolescents may choose not to have sex until they are older. Parents and adolescents look through magazines to identify messages about adolescent sexuality and discuss reasons for and against having sex.
6	Parents teach their children assertiveness skills. Parents ask their children questions to help them think about what it means when someone says "no" and how to respect that person's decision. Parents and adolescents come up with reasons why adolescents who are engaging in sexual activity choose to use condoms and why they might not want to use condoms.
7	Parents and adolescents read scenarios and respond to questions about what they would say or do, how they would be assertive, and how they would remain abstinent in various sexual situations. Parents and adolescents read scenarios and respond to questions about how they would be assertive about using condoms in sexual situations. Parents and adolescents play "The Condom Game," in which each step of correct condom use is written on a card, and both put the cards in the correct order.
8	Parents identify the most important thing they learned in the program, the skills they feel most comfortable using, and the skills that are most difficult for them. Parents make a list of the weekly activities that they were unable to complete and make a plan to do these activities in the future. Parents identify the next sex-related conversation they want to have with their child and set a timeline for that conversation.

## References

[1] Grunbaum JA, Kann L, Kinchen S, Ross J, Hawkins J, Lowry R (2004). Youth risk behavior surveillance — United States, 2003. MMWR Surveill Summ.

[2] Pedlow CT, Carey MP (2003). HIV sexual risk-reduction interventions for youth: a review and methodological critique of randomized controlled trials. Behav Modif.

[3] Johnson BT, Carey MP, Marsh KL, Levin KD, Scott-Sheldon LA (2003). Interventions to reduce sexual risk for the human immunodeficiency virus in adolescents, 1985–2000: a research synthesis. Arch Pediatr Adolesc Med.

[4] Kirby D (2001). Emerging Answers: research findings on programs to reduce teen pregnancy. Washington (DC): National Campaign to Prevent Teen Pregnancy.

[5] Jemmott JB, Jemmott LS, Fong GT (1998). Abstinence and safer sex HIV risk-reduction interventions for African American adolescents: a randomized controlled trial. JAMA.

[6] Capaldi DM, Crosby L, Stoolmiller M (1996). Predicting the timing of first sexual intercourse for at-risk adolescent males. Child Dev.

[7] Sieverding JA, Adler N, Witt S, Ellen J (2005). The influence of parental monitoring on adolescent sexual initiation. Arch Pediatr Adolesc Med.

[8] DiLorio C, Dudley WN, Soet JE, McCarty F (2004). Sexual possibility situations and sexual behaviors among young adolescents: the moderating role of protective factors. J Adolesc Health.

[9] Miller KS, Forehand R, Kotchick BA (1999). Adolescent sexual behavior in two ethnic minority samples: the role of family variables. J Marriage Fam.

[10] Rogers KB (1999). Parenting processes related to sexual risk-taking behaviors of adolescent males and females. J Marriage Fam.

[11] Huebner AJ, Howell LW (2003). Examining the relationship between adolescent sexual risk-taking and perceptions of monitoring, communication, and parenting styles. J Adolesc Health.

[12] DiClemente RJ, Wingood GM, Crosby R, Sionean C, Cobb BK, Harrington K (2001). Parental monitoring: association with adolescents' risk behaviors. Pediatrics.

[13] Bingham CR, Crockett LJ (1996). Longitudinal adjustment patterns of boys and girls experiencing early, middle, and late sexual intercourse. Dev Psychol.

[14] Gottfredson DC, McNeil RJ, Gottfredson GD (1991). Social area influences of delinquency: a multilevel analysis. Journal of Research in Crime and Delinquency.

[15] Dishion TJ, Patterson GR, Reid JR (1988). Parent and peer factors associated with drug sampling in early adolescence: implications for treatment. NIDA Res Monogr.

[16] Resnick MD, Bearman PS, Blum RW, Bauman KE, Harris KM, Jones J (1997). Protecting adolescents from harm. Findings from the National Longitudinal Study on Adolescent Health. JAMA.

[17] Jaccard J, Dittus PJ, Gordon VV (1996). Maternal correlates of adolescent sexual and contraceptive behavior. Fam Plann Perspect.

[18] Crosby RA, Miller KS, Wingood GM, DiClemente RJ (2002). Family influences on adolescent females' sexual health. Handbook of women's sexual and reproductive health.

[19] Tucker SK (1989). Adolescent patterns of communication about sexually related topics. Adolescence.

[20] Casper LM (1990). Does family interaction prevent adolescent pregnancy?. Fam Plann Perspect.

[21] Furstenberg FF, Herceg-Baron R, Shea J, Webb D (1984). Family communication and teenagers' contraceptive use. Fam Plann Perspect.

[22] Hutchinson MK, Jemmott JB, Jemmott LS, Braverman P, Fong GT (2003). The role of mother–daughter sexual risk communication in reducing sexual risk behaviors among urban adolescent females: a prospective study. J Adolesc Health.

[23] Karofsky PS, Zeng L, Kosorok MR (2000). Relationship between adolescent-parental communication and initiation of first intercourse by adolescents. J Adolesc Health.

[24] DiClemente RJ, Wingood GM, Crosby R, Cobb BK, Harrington K, Davies SL (2001). Parent-adolescent communication and sexual risk behaviors among African American adolescent females. J Pediatr.

[25] McNeely C, Shew ML, Beuhring T, Sieving R, Miller BC, Blum RW (2002). Mothers' influence on the timing of first sex among 14- and 15-year-olds. J Adolesc Health.

[26] Lefkowitz ES, Sigman M, Au TK (2000). Helping mothers discuss sexuality and AIDS with adolescents. Child Dev.

[27] Jaccard J, Dittus PJ, Gordon VV (2000). Parent-teen communication about premarital sex: factors associated with the extent of communication. J Adolesc Res.

[28] DiIorio C, Resnicow K, Thomas S, Wang DT, Dudley WN, Van Marter DF (2002). Keepin' it R.E.A.L.!: program description and results of baseline assessment. Health Educ Behav.

[29] Raffaelli M, Bogenschneider K, Flood MF (1998). Parent-teen communication about sexual topics. J Fam Issues.

[30] Romo LF, Nadeem E, Au TK, Sigman M (2004). Mexican-American adolescents' responsiveness to their mothers' questions about dating and sexuality. J Appl Dev Psychol.

[31] Cappello D (1997). Plain talk training package.

[32] Cyprian J (1998). Teaching human sexuality: a guide for parents and other caregivers.

[33] Tiffany JS, Tobias D, Raqib A, Ziegler J (1993). Talking with Kids about AIDS Resource Manual and Teaching Guide.

[34] Davis SL, Koblinsky SA, Sugawara AI (1986). Evaluation of a sex education program for parents of young children. J Sex Educ Ther.

[35] Caron SL, Knox CB, Rhoades C, Aho J, Tulman KK, Volock M (1993). Sexuality education in the workplace: seminars for parents. J Sex Educ Ther.

[36] Dittus P, Miller KS, Kotchick BA, Forehand R (2004). Why parents matter!: the conceptual basis for a community-based HIV prevention program for the parents of African American youth. J Child Fam Stud.

[37] Kirby D, Miller BC (2002). Interventions designed to promote parent-teen communication about sexuality. New Dir Child Adolesc Dev.

[38] Felner RD, Brand S, Mulhall KE, Counter B, Millman JB, Fried J (1994). The parenting partnership: the evaluation of a human service/corporate workplace collaboration for the prevention of substance abuse and mental health problems, and the promotion of family and work adjustment. J Prim Prev.

[39] Forehand R, Middlebrook J, Rogers T, Steffe M (1983). Dropping out of parent training. Behav Res Ther.

[40] Schuster MA, Eastman KL, Fielding JE, Rotheram-Borus MJ, Breslow L, Franzoi LL (2001). Promoting adolescent health: worksite-based interventions with parents of adolescents. J Public Health Manag Pract.

[41] Winick C, Rothacker DQ, Norman RL (2002). Four worksite weight loss programs with high-stress occupations using a meal replacement product. Occup Med (Lond).

[42] Hutter H, Moshammer H, Neuberger M (2006). Smoking cessation at the workplace: 1 year success of short seminars. Int Arch Occup Environ Health.

[43] Eastman KL, Corona R, Ryan GW, Warsofsky AL, Schuster MA (2005). Worksite-based parenting programs to promote healthy adolescent sexual development: a qualitative study of feasibility and potential content. Perspect Sex Reprod Health.

[44] Fishbein M, Bandura A, Triandis HC, Kanfer FH, Becker MH, Middlestadt SE (1991). Factors influencing behavior and behavior change: final report to the Theorist's Workshop.

